# Sex-specific differences in primary Sjögren's disease

**DOI:** 10.3389/fdmed.2023.1168645

**Published:** 2023-05-16

**Authors:** Achamaporn Punnanitinont, Jill M. Kramer

**Affiliations:** Department of Oral Biology, School of Dental Medicine, The University at Buffalo, State University of New York, Buffalo, NY, United States

**Keywords:** X chromosome, estrogen, X chromosome inactivation (XCI), microbiome, salivary glands

## Abstract

Many autoimmune diseases show a striking female sex predilection, including primary Sjögren's disease (pSD). Patients with pSD display exocrine gland pathology, such as salivary hypofunction and salivary and lacrimal gland inflammation. Moreover, many serious systemic disease manifestations are well-documented, including interstitial nephritis, hypergammaglobulinemia and neuropathies. Of note, women and men with pSD display distinct clinical phenotypes. While the underlying reasons for these clinical observations were poorly understood for many years, recent studies provide mechanistic insights into the specific regulatory landscapes that mediate female susceptibility to autoimmunity. We will review factors that contribute to the female sex bias, with an emphasis on those that are most relevant to pSD pathogenesis. Specifically, we will focus on sex hormones in disease, genetic alterations that likely contribute to the significant disease prevalence in females, and studies that provide evidence for the role of the gut microbiota in disease. Lastly, we will discuss therapeutics that are in clinical trials for pSD that may be particularly efficacious in targeting signaling networks that mediate inflammation in a sex-specific manner.

## 1. Introduction

Autoimmune diseases consist of more than 70 chronic disorders, which affect approximately 5% of the US population (1). Many autoimmune diseases preferentially affect females, and the most striking sex differences are observed in Sjögren's disease (pSD), also referred to as Sjögren's syndrome), systemic lupus erythematosus (SLE), autoimmune thyroid disease and scleroderma, which represent a spectrum of diseases in which the patient population is at least 80% women (1). Our understanding of the molecular basis underlying this sexual dimorphism is becoming increasingly sophisticated, and this review will discuss the current knowledge with a focus on sex-specific molecular mechanisms that are particularly relevant to pSD pathogenesis.

SD occurs in 2 forms: primary (pSD) and secondary (sSD). In pSD, SD is the only autoimmune disease present. In contrast, sSD is observed in conjunction with another autoimmune connective tissue disease (2). This review will focus on the primary form of the disease, as pSD shares overlapping features with other autoimmune diseases, and it is often difficult to determine which disease manifestations are caused solely by SD in sSD studies (3). The estimated prevalence of pSD is 0.3–1.0 per 1,000 persons (4). Worldwide, the estimated prevalence of pSD ranges between 0.01 and 0.72% (5). Primary SD exhibits a striking female predilection. Indeed, a recent clinical study estimated that the age- and sex-adjusted prevalence of pSD in the US was 16.3 per 10,000 in women and 3.1 per 10,000 in men (6).

The current classification criteria for pSD were validated jointly by the American College of Rheumatology and the European League against Rheumatism (7). These criteria include the assessment of anti-Ro/SSA autoantibodies, focal lymphocytic sialadenitis (FLS), and evaluation of salivary and ocular function (7). Each of these manifestations is scored and the presence of anti-Ro/SSA antibodies and FLS are weighted most heavily (7). In addition to these disease manifestations, patients may also experience interstitial lung disease and nephritis (8). Moreover, pSD patients display systemic hematopoietic abnormalities including hypergammaglobulinemia and hypocomplementemia, and patients are at increased risk of developing B cell lymphoma (2, 9, 10). Dryness of the mouth and eyes, fatigue and joint pain are common symptoms found in greater than 80% of patients with pSD, and these disease manifestations have a debilitating effect on the patient's quality of life (8). In addition to the significant morbidity associated with the disease, pSD patients also showed increased risk of mortality. A recent meta-analysis that included 14 studies with 14,584 pSD patients reported a total of 902 deaths (11). Compared to the general population, pSD patients had a 1.46-fold increased risk of death (11). Several factors contributed to the increased risk of mortality, including male sex, older age, hypocomplementemia, and interstitial lung disease (11).

Despite the significant morbidity and mortality associated with pSD, the underlying disease mechanisms that govern pSD remain poorly understood and the signaling networks that control sex-specific disease manifestations are not fully elucidated. This review will provide an overview of etiologic factors that likely contribute to the female sex bias in pSD. Herein, we will briefly summarize clinical data from pSD patients that highlight differences in disease presentation between males and females. Next, we will investigate evidence showing the role of sex hormones in pSD and examine findings detailing sex-specific genetic alterations that may contribute to pSD. We will then discuss how microbiota may mediate pSD. Lastly, we will discuss emerging therapeutics that may be tailored to pSD patients in a sex-dependent manner. This topic carries important clinical relevance, as it is crucial to decipher the networks that mediate pSD to identify treatment strategies that are relevant to patient sex.

## 2. Male and female pSD patients display distinct disease manifestations

For reasons that remain incompletely understood, males and females with pSD tend to display distinct disease manifestations (12). In a recent clinical study that identified comorbidities of pSD patients according to age and sex, female pSD patients had an increased risk of developing numerous conditions, such as fibromyalgia, migraines, hypermobile syndromes, Ehlers-Danlos syndrome and CREST syndrome, while men with pSD were at an increased risk of developing cerebrovascular accidents and cardiac pathologies such as myocardial infarction, atherosclerosis, and congestive heart failure (12). It is important to note that this study did not compare pSD patient data to that from healthy control subjects, and thus it is unclear whether these comorbidities are associated solely with patient sex, or whether they are related specifically to pSD. Further studies to determine whether these comorbidities are increased in pSD patients in a sex-specific manner will be insightful.

Although men tend to develop autoimmune diseases less commonly than women, men often exhibit more severe disease outcomes. In a separate study that included 967 patients with pSD (899 females and 68 males), men with pSD were more frequently diagnosed with interstitial lung disease, lymphadenopathy and lymphoma, while hypothyroidism was more common among females with pSD (13). Similarly, a corroborative study in which medical records of 353 female and 33 male pSD patients were reviewed retrospectively found that pulmonary complications and lymphoma were seen more frequently in males, while females had a higher prevalence of autoimmune thyroid diseases (14).

While sex-specific clinical manifestations tend to be consistent between studies, disparate serological findings are reported. Indeed, while one study reported higher anti-Ro/SSA antibodies in female pSD patients (13), another revealed that males displayed positivity for anti-Ro/SSA, anti-La/SSB and anti-nuclear autoantibodies (ANAs) more frequently than women (14). Such disparities may be explained by the low male prevalence in patient cohorts or by the different methods used to detect serum autoantibodies, as one group used double immunodiffusion while the other employed ELISAs (15). A comprehensive review of the clinical differences between males and females with pSD was recently published and provides an excellent overview of the topic (12).

## 3. Estrogen mediates a protective effect in exocrine tissue in pSD

Since women are primarily affected by pSD, there has been considerable interest in understanding the role of estrogen and estrogen precursors in disease and several studies have been performed in mouse models to establish a role for sex hormones in pSD. Indeed, healthy ovariectomized C57BL/6 mice exhibited an exocrinopathy with autoimmune characteristics resembling pSD, including lymphocytic infiltration of salivary and lacrimal glands, and anti-Ro/SSA, anti-La/SSB, and anti-α-fodrin autoantibodies (16). Additional elegant work revealed that estrogen deficiency in mice induced the expression of the retinoblastoma-associated protein 48 (RbAp48) in exocrine glands (17). Transgenic (Tg) expression of RbAp48 in the exocrine glands caused estrogen-dependent apoptosis (17). Specifically, overexpression of RbAp48 induced p53-mediated apoptosis in both salivary and lacrimal tissue of mice lacking estrogen, while inhibition of RbAp48 expression inhibited this apoptosis (17).

In a corroborative study, Tg mice overexpressing RbAp48 under the control of exocrine-specific parotid secretory protein promoter exhibited SD-like exocrinopathy characterized by an increase in apoptosis. In addition, lacrimal and salivary gland epithelial cells produced heightened IFNγ and IL-18 in this model (18, 19). Furthermore, salivary gland epithelial cells (SGECs) derived from RbAp48-Tg mice expressed elevated levels of MHC class II and the costimulatory molecules CD86, CD80, and ICAM-1 (18). Importantly, studies in human salivary cells found that both estrogen receptor α (ERα) and ERβ were expressed and functional (20). Accordingly, treatment of SGECs with estrogen resulted in decreased IFNγ-inducible expression of ICAM-1 (20). These data demonstrate that estrogen protects against exocrine gland apoptosis, and diminished estrogen promotes a pro-inflammatory phenotype in salivary tissue.

Corroborative work in the aromatase knockout (ArKO) mouse model also supports a protective role for estrogen in exocrine tissue. Aromatase is an enzyme that converts androgens to estrogens, and thus ArKO mice are deficient in estrogen (21). ArKO mice developed a lymphoproliferative autoimmune disease resembling SD (21, 22), as histological analyses showed inflammatory infiltrates in the lacrimal and salivary glands of ArKO mice that increased with age (21). Administration of an aromatase inhibitor resulted in enhanced lymphocytic infiltration in both salivary and lacrimal tissue derived from the NFS/*sld* mouse model of SD (21). These data provide further evidence for the protective role of estrogen in the attenuation of immune activation in exocrine tissue.

The role of estrogen in pSD has also been assessed in female patients. A recent case-control study of 1,320 pSD patients and 1,360 sicca controls examined estrogen exposure and disease risk (23). In contrast to murine findings, this study found that greater lifetime estrogen exposure is not necessarily protective against disease. Indeed, composite estrogen scores of pSD patients, which was a score assigned based on estimated lifetime estrogen exposure, did not correlate with unstimulated whole saliva production, Schirmer's test scores, or salivary focus scores (23). The authors also analyzed cumulative menstrual cycling (CMC) as a separate surrogate marker of estrogen exposure, which was calculated as years menstruating minus time pregnant. A higher CMC score was associated with greater unstimulated whole salivary production, although ocular staining score, hypergammaglobulinemia, and salivary focus score were inversely associated with CMC score in pSD patients (23). Thus, the data remain somewhat inconclusive as to whether estrogen exposure is protective or pathogenic in pSD patients, and additional longitudinal studies are needed to demonstrate this conclusively.

## 4. Altered expression of X-chromosome genes likely contributes to pSD

Although the underlying mechanisms that mediate the female sex predilection in autoimmunity are not completely understood, it is clear that altered expression of X chromosome alleles has a significant impact on disease susceptibility (24). Indeed, males with increased X chromosome copy number are more susceptible to autoimmune disease, as male patients with Klinefelter's syndrome (47, XXY) have a 38-fold increase in the incidence of pSD as compared to healthy men (25). Further evidence of the role of the X chromosome in autoimmunity is provided by one study reporting a 16-year-old Japanese patient with trisomy X (47, XXX) who developed mixed connective tissue disease and SD (26). In addition, a separate study found trisomy X women have a 2.9-fold higher risk of developing pSD as compared to females lacking the trisomy phenotype (46, XX) (27). These studies demonstrate the pathogenic potential of overexpression of X chromosome-related genes in pSD.

Of direct relevance to these observations, X chromosome inactivation (XCI) is the primary regulatory mechanism of X-linked gene dosage compensation between males and females. In XCI, one of the X chromosomes in female cells is transcriptionally silenced by epigenetic mechanisms in a stochastic manner, leading to allele-specific enrichment of epigenetic modifications on the inactive X (Xi). XCI ensures that X-linked genes are expressed at similar levels between females and males (28). In theory, this dosage compensation is stably maintained with each cell division in all female somatic cells (29). It is important to note that many genes associated with immunity are expressed on the X chromosome, including *TLR7*, *TLR8*, *BTK*, and CD40 ligand (*CD40LG*).

While this mechanism is generally effective at maintaining equivalent expression of X chromosome genes between the sexes, it is estimated that approximately 20% of X-linked human genes escape XCI (30). This inefficiency results in leaky expression of some X-linked genes from the Xi known as XCI escape, and this has important implications for autoimmune diseases, particularly those that display a female predilection. XCI is mediated by the long non-coding RNA termed XIST. It was initially thought that XIST established XCI early in development and then was no longer required to regulate gene expression in the female cells. Recent elegant work, however, established that XIST is crucial in maintaining XCI continually for specific X-linked genes in human B cells, including TLR7 (31).

This has important implications for autoimmunity, as XCI maintenance is altered in both B and T cells of SLE patients and mice, and X-linked genes are upregulated in adaptive immune cells from SLE patients as compared to healthy controls (28, 32). While XCI escape has not been identified in pSD patients thus far to our knowledge, these studies suggest that altered expression of particular X-linked genes could contribute to increased disease prevalence observed in females with pSD. Indeed, numerous genes that reside on the X chromosome are implicated in pSD, and differential expression and methylation of genes associated with immune activation are reported both in salivary tissue and peripheral blood cells in pSD patients (33). An overview of studies related to the expression and function of these genes is provided below:

*TLR7*: TLR7 is an endosomal TLR that is encoded on the X chromosome. An extensive body of literature demonstrates that TLR7 activation is pathogenic in the context of lupus (34–36), and emerging studies indicate that TLR7 may also mediate pathology in pSD. TLR7 is increased and functional in salivary tissue and in peripheral blood cells of pSD patients (37–41) and data suggest B cells and monocytes are hyper-responsive to TLR7 agonism in pSD (42–45). Indeed, TLR7 stimulation of B cells from pSD patients resulted in enhanced plasma cell differentiation and elevated IFNα secretion (42, 44).

Data from mouse models also provide corroborating evidence that TLR7 agonism mediates organ-specific disease (46–48). Studies in *TLR8−/−* animals revealed that these mice develop lupus and SD concomitantly, as sialadenitis, autoantibody production, glomerulonephritis, and lung inflammation were observed (46, 49). These disease manifestations were dependent on TLR7, as disease was abrogated in mice lacking both TLR7 and TLR8 (46, 49). Further work in the NOD/ShiLtJ model revealed that TLR7 is required for lacrimal gland inflammation that is characteristic of SD (47). Finally, corroborative work by our group revealed that TLR7 agonism accelerates both local and systemic disease in a mouse model of pSD and drives the expansion of a subset of B cells that are pathogenic in the context of lupus, termed age-associated B cells or ABCs (50).

Alterations in TLR7 expression and function are observed even in healthy 46, XX women (51). Indeed, TLR7 escapes XCI and is overexpressed in plasmacytoid DCs (pDCs), monocytes and B cells from women and biallelic expression of TLR7 was observed in the majority of donors tested (51). Moreover, B cells that showed biallelic expression of TLR7 showed a 2-fold greater likelihood of undergoing class switching as compared to monoallelic B cells (51). Thus, expression of TLR7 from both alleles could result in enhanced plasma cell differentiation. These findings are relevant to pSD, as B cells derived from pSD patients are hyper-responsive to TLR7 ligation and pSD patients often display hypergammaglobulinemia and high autoantibody titers (9, 10, 42, 44). Additionally, *TLR7* expression was increased in CD19+ peripheral B cells derived from pSD female patients as compared to sex-matched healthy controls (52). Altogether, these studies suggest TLR7 likely contributes to the female disease predilection observed, although further studies are needed to establish whether high expression of TLR7 in immune cells mediates disease in the context of pSD.

In a corroborative study, XIST was found to be dysregulated in female patients with SLE, and this led to the XCI escape of *TLR7* (31). Additionally, TLR7 agonism resulted in the inactivation of XIST and promoted the expansion of ABCs (31). Of note, B cells with features of ABCs are documented in pSD patients as well, although XIST inactivation of the *TLR7* locus has not been reported in pSD patients or models to date (53, 54). Altogether, these data suggest that TLR7 dysregulation mediates pSD pathogenesis, and this may account, at least in part, for the striking female disease predilection observed. Table 1 provides a summary of studies that indicate altered expression and/or function of TLR7 in pSD mouse models and patients (37, 39–42, 44–48, 50, 52, 55).

**Table 1:** Evidence for TLR7 dysregulation in SD mouse models and patients.

	Summary of results	Ref
**Murine model**		
*TLR7−/−,* *TLR8**−/−* on C57BL/6 background	Systemic ablation of Tlr7 in a mouse model of lupus ameliorated SD-like disease manifestations in females	(46)
*TLR7−/−* NOD/ShiLtJ	Systemic ablation of Tlr7 diminished lacrimal gland inflammation in males	( 47 )
NOD.B10Sn-*H2^b^*	Treatment of female pSD mice accelerated local and systemic disease and promoted the expansion of ABCs	( 50 )
B6J.NOD/ShiLtJ-*Aec1Aec2*	TLR7 signaling was implicated in defective efferocytosis in bone marrow-derived macrophages derived from pSD mice	( 48 )
**Human tissue**		
Minor salivary glands	SD female patients (*n* = 40) had elevated *TLR7* expression as compared to non-SD controls (*n* = 11)	( 46 )
	Salivary ducts, B cells, plasma cells and pDCs expressed TLR7 in female pSD patients (*n* = 10)	( 41 )
	TLR7 was expressed in lymphocytic infiltrates in pSD patient biopsies	( 40 )
Parotid glands	TLR7 was expressed in salivary ducts and infiltrating lymphocytes in pSD tissue (*n* = 17 female/3 male). TLR7 was expressed in salivary ducts in healthy control (HC) tissue (*n* = 8 female/ 2 male)	( 37 )
PBMCs	TLR7 signaling pathways were dysregulated in female pSD patients (*n* = 25) as compared to HCs (*n* = 25)	( 45 )
	Cultured PBMCs from female pSD patients secreted IFNα in response to IFNβ treatment (*n* = 6)	( 41 )
	*TLR7* expression was increased in PBMCs from pSD patients (*n* = 36 female/ 1 male) as compared to HCs (*n* = 23 female/ 1 male)	( 37 )
	PBMCs from pSD patients (*n* = 18 female/2 male) had elevated TLR7 expression as compared to female HCs (*n* = 20)	( 39 )
B cells	Treatment of naïve B cells from female pSS patients (n = 14) with a TLR7 agonist results in increased class switching and plasma cell differentiation as compared to HCs (*n* = 18)	( 44 )
	TLR7 agonism increased IFNα secretion in B cells derived from female pSD patients (*n* = 21) as compared to HCs (*n* = 18)	( 42 )
	*TLR7* was increased in CD19^+^ B cells from Ro-positive female pSD patients (*n** *= 12) as compared to sex-, age-, and ethnicity-matched HCs (*n** *= 20)	( 52 )
pDCs	*TLR7* was upregulated in IFN-positive pDCs from pSD patients (*n* = 9) as compared to IFN-negative pSD patients (*n* = 7) and HCs (*n* = 7)	( 40 )
CD14+ monocytes	*TLR7* was upregulated in IFN-positive monocytes from pSD patients (n = 50) as compared to IFN-negative pSD patients (*n* = 50) and HCs (*n* = 41)	( 40 )
Monocytic DCs	Monocytic DCs from pSD patients (*n* = 8) matured for 48 h with TLR7/8 ligand CL097 expressed significantly less Stat1 than those derived from HCs (*n* = 8)	( 55 )

*CXorf21*: Chromosome X open reading frame 21 (*CXorf21*) (also called *TASL*) is a risk allele for both SLE and pSD (56). *CXorf21* is expressed by pDCs, B cells, and CD14+ and CD16+ monocytes (57, 58). CXorf21 serves as a cytosolic adapter molecule for TLR7, and its activation culminates in the production of IFNα (56). To test the function of CXorf21 in female and male immune cells, knockdown of *CXorf21* was performed in primary human heathy monocytes. TLR7 stimulation of female monocytes induced *CXorf21* expression, and knockdown of *CXorf21* abrogated this increase (58). In contrast, expression of *CXorf21* in male monocytes was negligible, and there was no change in relative expression of *CXorf21* following knockdown and subsequent TLR7 stimulation (58). Moreover, in healthy female monocytes in which knockdown of *CXorf21* was carried out*,* TLR7 stimulation resulted in a 7-fold decrease in *IFNA1* compared with sham-treated control cells (58). Finally, studies in lymphoblastoid cell lines (LCLs) derived from SLE patients revealed expression of *CXorf21* was increased in 47,XXX/46,XX women and 47,XXY/46,XY men compared to LCLs derived from healthy males (58).

Additional studies by the same group revealed that CXorf21 escapes XCI and is therefore expressed at higher levels in healthy human female cells as compared to those derived from males, and female monocytes had a more acidic lysosomal pH as compared to males (56). This is significant because TLR7 is cleaved to its active form by a mechanism that is initiated by a low endosomal pH (59). This decreased pH may be of clinical significance because this alteration could contribute to the hyper-responsive endolysosomal-dependent immune response that is seen in females as compared to males (56), and this could contribute to the heightened TLR7 and IFN responses that are observed in women, although further studies are needed to establish whether this mechanism contributes to enhanced immune activation in female pSD patients.

*BTK*: BTK is expressed on the X chromosome and is also implicated in pSD, although no studies, to our knowledge, have demonstrated that BTK escapes XCI. BTK is activated upon B cell receptor stimulation and thus plays a crucial role in B cell activation and development (60). BTK expression was increased in circulating B cells from pSD patients with high serum autoantibodies and this correlated with T cell infiltration in parotid tissue (61). Overexpression of BTK in B cells specifically drives disease that is reminiscent of both SLE and pSD in mice (62, 63). Finally, *BTK* was identified as a key driver gene in salivary tissue from pSD patients (64).

*CXCR3*: CXCR3 is a chemokine receptor that binds CXCL9 and CXCL10. CXCR3 is expressed by activated CD4+ and CD8+ T cells, and functions to promote recruitment of Th1 cells in an immune response (65). *CXCR3* exhibits XCI, as T cells derived from female SLE patients show elevated expression of *CXCR3* as compared to those from males (66). In pSD patients, elevated levels of CXCL9 and CXCL10 are observed in salivary tissue, and infiltrating CXCR3+ T cells correlate with levels of these chemokines (67). Moreover, CXCR3 is expressed constitutively by salivary epithelial cells derived from both pSD patients and non-SD controls, although it is functionally impaired in tissue derived from pSD patients (68). *CXCR3* expression was also increased in the conjunctiva of patients with pSD as compared to controls (69).

*CD40LG: CD40LG* encodes CD40L, an activation marker that is expressed by T cells. CD40L engages CD40 on B cells and other antigen-presenting cells, and this interaction is required for germinal center formation (70). There is considerable evidence that CD40-CD40L interactions are enhanced in pSD. Recent transcriptome analyses of salivary gland tissue derived from patients with pSD revealed a CD40 signaling gene signature that was enriched in B cells (71). Moreover, CD40L expression was increased on activated CD4+ T cells in patients with pSD and sera derived from pSD patients exhibited elevated levels of soluble CD40L as compared to that from healthy controls (72, 73). Of relevance to pSD, *CD40LG* was demethylated in T cells derived from female SLE patients, and this correlated with enhanced *CD40LG* expression in T cells derived from females with lupus as compared to males with the disease (74). While this heightened expression of *CD40LG* likely contributes to immune activation in SLE and pSD, the functional consequences of this have yet to be established.

In addition to genes encoded by the X chromosome, several genes that show sex-biased expression are implicated in pSD pathogenesis. One such gene is Vestigial-like family member 3 *(VGLL3*), a cofactor of the TEA-domain containing transcription factor (TEAD) that contributes to diverse inflammatory processes such as autoimmunity (75). While *VGLL3* is not located on the X chromosome, this gene is expressed at higher levels in women in both the skin and parotid gland (76). Complement genes are integral to immune function and *C4A* and *C4B* display sexual dimorphism (77). Alleles that increase gene dosage of *C4A* strongly protect against SLE and pSD. Effects of *C4* alleles are stronger in men with SLE as compared to female SLE patients, although similar analyses performed in patients with pSD had limited power due to the small numbers of male pSD patient samples available (77). Additional genes display sexual dimorphism in murine salivary tissue in pSD that could contribute to disease, such as those that encode IL-17 and Kallekrein protease family members (78, 79).

**Figure 1:**
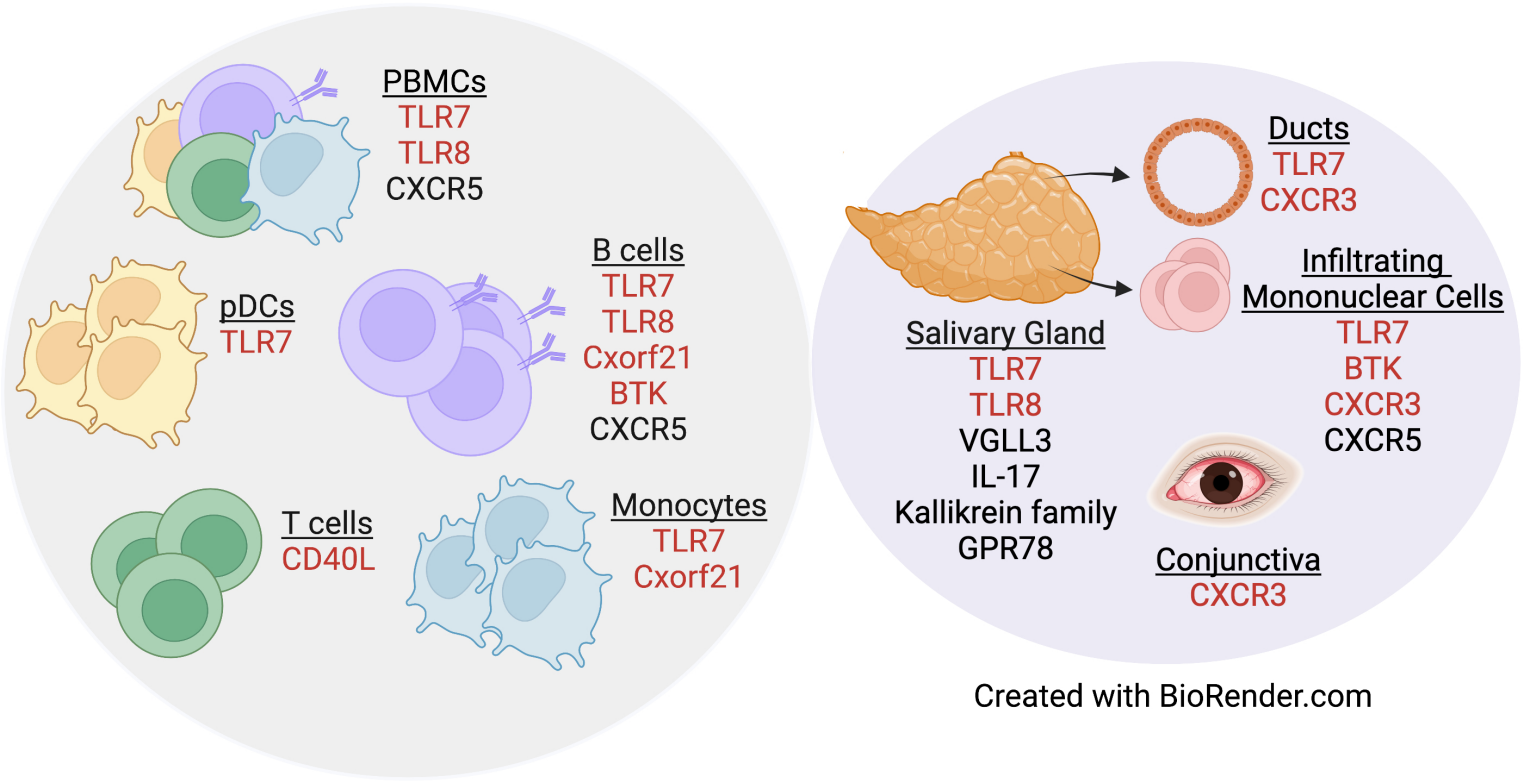
Genes dysregulated in pSD pathogenesis are differentially expressed in a sex-specific manner. Both peripheral (gray circle) and exocrine tissue (purple circle) show alterations in sexually dimorphic genes in pSD. Red text indicates genes that are expressed on the X chromosome.

A separate study that examined primary B cells from males and females reported that *CXCR5* is a pSD-associated SNP that displayed differential eQTL effects in women as compared to men (80). Dysregulation of CXCR5 is well-established in pSD, as CXCR5 expression was increased in lymphocytes within salivary tissue but decreased on peripheral B and T cells (81). Additionally, IRF5 is expressed in a sex-biased manner, as *IRF5* gene expression was upregulated in splenic tissue derived from both healthy and lupus-prone females as compared to strain-matched males (82). Of relevance to pSD, polymorphisms in *IRF5* are associated with pSD (83, 84) and TLR7 activation of IRF5 mediates the production of type I IFNs (85). Finally, recent data demonstrate that G protein receptor 78 (GPR78) is elevated in minor salivary gland tissue from male pSD patients as compared to females with pSD and male healthy controls (86). Interestingly, a mouse model that overexpressed GPR78 displayed salivary hypofunction and enhanced epithelial apoptosis, and these findings were observed in males but not females (87). Figure 1 contains a summary of genes dysregulated in pSD that are related to the female sex bias observed and a summary of genes implicated in human SLE and pSD is provided in Table 2.

**Table 2:** A comparison of genes with sex-biased expression in SLE and pSD.

Gene	Human disease association		Refs
	SLE	pSD	
**X-linked**			
*TLR7*	• Increased in immune cells from females with SLE compared to males with SLE and HC females • *TLR7* polymorphisms associated with SLE	• Increased in immune cells and salivary tissue from pSD patients compared to healthy controls (HCs)	Table 1 (31, 88–90)
*TLR8*	• *TLR8* polymorphisms are associated with SLE	• Increased in CD19^+^ B cells from Ro-positive pSD females compared to female HCs	(52, 88, 90)
*CXorf21*	• Risk allele for SLE • Elevated in immune cells from female and male SLE patients compared to sex-match HCs	• Risk allele for pSD	(56, 57)
*BTK*	• Elevated in PBMCs from SLE patients compared with HCs • BTK+ B cells elevated in SLE patients with lupus nephritis (LN) compared to LN-negative SLE patients	• Increased in B cells from pSD patients compared to HCs	(61, 91, 92)
*CXCR3*	• Increased in female SLE T cells compared to those from males with SLE	• Increased in conjunctiva of pSD patients compared to HCs	(66, 69)
*CD40LG*	• Increased in female SLE T cells compared to those from males with SLE	• Increased in activated CD4+ T cells from female pSD patients compared to HCs	(72, 74)
**Sex-biased expression, non-X-linked**			
*VGLL3*	• Increased and localized to the nucleus in skin of HC females and SLE patients of both sexes	• Increased in parotid tissue from pSD patients compared to HCs	( 76 )
*C4A, C4B*	• Alleles that increase gene dosage of *C4A* strongly protect against SLE • Effects of *C4* alleles are stronger in men with SLE	• Alleles that increase gene dosage of *C4A* strongly protect against pSD	(77, 93)
*CXCR5*	• Expression is increased in CD4+ T cells from female SLE patients compared to sex-matched HCs	• Risk allele for pSD • Differentially regulated in pSD females compared to males with pSD	(80, 83, 94, 95)
*IRF5*	• Risk allele for SLE	• Risk allele for pSD	(83, 95, 96)
*GPR78*	• Not reported	• Elevated in minor salivary gland tissue from pSD male patients compared to female pSD patients and male HCs	( 86 )

## 5. Estrogen governs expression of genes implicated in autoimmunity

Recent work highlights the complex regulatory network that exists between estrogen and immune-related genes, including those expressed on the X chromosome. Indeed, elegant studies in a humanized mouse model revealed that TLR7 responses are enhanced in female pDCs as compared to those derived from males (87). Moreover, treatment of post-menopausal women with 17β-estradiol, the main estrogenic hormone, resulted in elevated IFNα secretion by pDCs in a TLR7-dependent manner (87). When ERα was ablated in the DC lineage, this population was rendered unresponsive to TLR7-dependent Type I IFN production (87). Additionally, estrogen stimulation of CD11b+ monocytes derived from C57BL/6 female mice culminated in the enhanced expression of *Unc93b1*, a molecular chaperone required for TLR7 activity (97) and splenocytes from lupus-prone female mice expressed higher levels of *Unc93b1* as compared to C57BL/6 controls (98).

Moreover, splenocytes derived from ERα-deficient mice expressed decreased *Irf5* when compared to those from ERα-sufficient controls, and treatment of splenocytes with 17β-estradiol resulted in increased *Irf5* levels (82). In addition, mice lacking expression of ERα in either the DC or hematopoietic compartment showed reduced frequency of Type I IFN–producing pDCs following TLR7 stimulation (99). Finally, studies in humans demonstrate levels of IRF5 and ERα correlate in pDCs derived from females but this association is not observed in male pDCs (99). It is important to point out that studies in the peripheral blood suggest that estrogen is pro-inflammatory, while studies in salivary tissue indicate a protective role for this hormone in the context of pSD, and epidemiologic data from pSD patients is somewhat inclusive at present (*vide supra*). This dichotomous role of estrogen highlights the complex influence of sex hormones in autoimmunity and suggests both pro-inflammatory and protective effects of sex hormones, depending on the cell type and specific microenvironment. Collectively, these data establish mechanisms whereby estrogen exerts potent effects on the expression of genes that are dysregulated in autoimmunity in a sex-dependent manner.

## 6. Sex-specific differences in microbiota may contribute to pSD

While still an emerging area of study, there are several lines of evidence that suggest sex steroids drive distinct differences in the composition of the intestinal microbiome and this has significant implications for health and disease. In both men and women, levels of sex hormones correlate with microbial diversity, and those with the highest levels of hormones show the greatest diversity (100). Significant differences are reported in the composition of the gut microbiome between pre- and post-menopausal women, as pre-menopausal women had increased levels of Bacteroidetes and Firmicutes species, which was reminiscent of the male microbiota (101). It is important to point out that while the human microbiome is shaped by biological sex and sex steroids, there is considerable variability among studies and the underlying mechanisms governing intestinal dysbiosis in this context remain incompletely understood (101).

While factors that shape the human microbiota are complex, seminal studies established an important sex-specific role for the microbiome in the autoimmune pathogenesis in mice (102, 103). Indeed, these studies revealed that microbial exposure early in life governs sex hormone levels and modifies disease progression in a mouse model of type I diabetes (T1D). Strikingly, when male mice of this strain were colonized with commensal microbiota, serum testosterone was elevated and the animals were protected from disease. When the microbiota from male mice was transferred to females of the same strain, the female recipients showed high testosterone levels and were protected from T1D (102). Transfer of specific species that comprise the microbiome can modulate disease in mice and humans. Indeed, corroborative work found that transfer of the intestinal bacteria *Blautia (Ruminococcus) gnavus* from SLE patients induced intestinal permeability that was enhanced in female recipient mice as compared to males. These recipient animals developed some features of SLE, such as anti-DNA autoantibodies (104). Of note, this effect was specifically related to patients with SLE, as transfer of the same bacteria derived from healthy donors did not induce this pathology (104). Additional work found that *Lactobacillus* strains protected against renal damage in the lupus-prone mouse model, MRL/*lpr*. Treatment of MRL/*lpr* mice with 5 strains of *Lactobacillus* resulted in reduced intestinal permeability and diminished IgG2a production, the antibody class that comprises most of the immune deposits observed in glomerulonephritis (105). Moreover, levels of IL-10 were increased, indicating a protective effect of these bacteria (105). Finally, these effects were sex-specific, as they were only observed in females and castrated males (105).

Additional mechanistic studies have uncovered a role for TLR7 activation in gut microbiota interactions in the context of autoimmunity. Indeed, TLR7 activation by intestinal bacteria resulted in lupus-like disease, as *Lactobacillus reuteri* mediated TLR7-dependent lupus in both conventional and germ-free mice (106). Complimentary studies in different lupus mouse models as well as in other female-biased autoimmune diseases, such as multiple sclerosis and rheumatoid arthritis (RA), provide additional evidence to support a role for sex-driven alterations in intestinal microbiota (107, 108).

While many studies demonstrate alterations in the gut, oral, and vaginal microbiome in patients with pSD (109), there is a paucity of data related to pathogenicity of the microbiota. There is one study, to our knowledge, that provides direct evidence for intestinal dysbiosis in a mouse model of pSD (110). Indeed, in studies in which gut microbiota from pSD patients was transferred to germ-free mice, the recipient animals developed heightened corneal barrier disruption (110). This study, however, only enrolled female pSD patients and microbiota was transferred to female mice, so additional work is needed to determine whether differences exist in male microbiota derived from pSD patients as compared to females, and whether alterations in the clinical phenotype occur between male and female recipient mice.

In addition, a separate study revealed that the bacteria identified in pSD patients were related to clinical disease features (109). Interestingly, treatment of pSD patients with hydroxychloroquine (HCQ) altered the composition of the microbiome in the oral cavity, vagina, and intestine, and this was distinct from that observed in healthy control subjects. Following 6–12 months of treatment, pSD patients who responded to HCQ treatment showed different oral microbiomes as compared to those derived from pSD patients who did not respond to therapy, and the microbiota of the responders was more similar to that of the healthy control subjects as compared to pSD patients who were refractory to treatment (109). Of note, HCQ suppresses inflammation through several mechanisms, including the prevention of endosomal acidification (111). Thus, one of the consequences of HCQ therapy could be the reduction of endosomal TLR activation, including TLR7 (112). However, HCQ has broad effects on many signaling networks (111), and additional studies are needed to determine if TLR7 governs or influences the microbiome in pSD in a sex-biased manner and whether this is of clinical consequence.

## 7. Implications for therapeutics

Altogether, these studies suggest that therapies targeting proteins derived from genes that are expressed in the X chromosome, particularly those that escape XCI, may be particularly efficacious in 46, XX females, 47, XXX females, and 47, XXY Klinefelter males. Drugs that target pathways that are overexpressed in a sex-biased manner may also have utility in these patient groups. There are several therapeutics in clinical trials that target products of genes that are expressed on the X chromosome.

### 7.1. Blockade of Cd40/CD40L interactions

Inhibition of CD40/CD40L interactions ameliorates pSD in mouse models, even when delivered at a pre-disease time point (113–115). Two drugs are currently in clinical trials for pSD that block this pathway: Dazodalibep and Iscalimab. Dazodalibep is an Fc-deficient CD40L antagonist fusion human monoclonal antibody. It binds to CD40L on activated T cells and blocks the interaction with CD40. Iscalimab is a humanized IgG1 monoclonal antibody directed against CD40. Both drugs are currently in phase 2 clinical trials for pSD (116–120).

### 7.2. BTK inhibition

Previous studies in both mice and humans provide a strong rationale for the use of therapies that target BTK in pSD. Indeed, deletion of BTK in a mouse model that has features of both pSD and lupus results in attenuated disease (121). Moreover, genetic analysis of salivary tissue from pSD patients revealed that BTK inhibition was likely to be an effective therapy for pSD patients with certain molecular signatures (64). A selective, covalent BTK inhibitor, Remibrutinib, is currently in phase 2 clinical trial in patients with moderate to severe pSD (122, 123).

### 7.3. TLR7 and TLR8 inhibition

Phase 2 clinical trials with Enpatoran, a highly selective small molecule inhibitor of TLR7 and TLR8, are ongoing for patients with SLE (124–126). There is considerable evidence in both mice and humans that TLR7 activation mediates immune dysfunction in pSD (Table 1), and thus there is a strong rationale to carry out clinical trials to assess the efficacy of this drug in pSD patients.

### 7.4. Drug efficacy differs between male and female patients with autoimmune disease

It is important to note that there are several studies with thousands of patients in aggregate that document differences in drug efficacy between males and females with autoimmune disease. This is observed most commonly in studies of male and female patients who receive biologic therapies. In patients with axial spondyloarthritis (315 females, 654 males), females showed a diminished response to first-line TNF inhibitors (TNFi) by the second year of treatment as compared to males (127) and females also exhibited a lower rate of disease remission (104 females, 236 males) (128). Moreover, a lower percentage of female patients with inflammatory arthritis (RA, ankylosing spondylitis, and psoriatic arthritis) achieved disease remission after TNFi therapy as compared to men (138 females, 167 males) (129). Similar findings are reported in patients with moderate to severe psoriasis, as female patients were less likely than males to achieve a therapeutic response after at least one year of TNFi therapy (83 females, 97 males) (130). Finally, a 48-week phase 3 randomized control trial of psoriatic arthritis patients found that women who received methotrexate and the TNFi etanercept had worse treatment outcomes as compared to men (139 females, 144 males) (131).

In addition, a study of RA patients (73 females, 28 males) revealed that in patients receiving the TNFi infliximab as a second-line drug, women were more likely to develop anti-drug antibodies and have lower serum infliximab levels as compared to males (132). Corroborative data were reported in inflammatory bowel disease (IBD) patients who received TNFi therapy for a minimum of 1 year, as females were more likely to discontinue the therapy than males, due to a higher incidence of side effects (265 females, 264 males) (133). Similar results were reported in patients with psoriasis, as females reported experiencing more side effects than males following biologic therapy, and these included fungal and herpes virus infections (127 females, 188 males) (134). Of note, contrasting findings were reported in patients with IBD, as males that received infliximab therapy showed greater anti-drug antibodies and lower concentrations of the drug in sera as compared to females (228 females, 233 males) (135). Interestingly, these observations were specific for infliximab, as there were no differences observed between male and female IBD patients who received the TNFi adalimumab (298 females, 333 males) (135). Altogether, these findings provide evidence that biologic therapies have different efficacies in men and women with autoimmunity, and biological sex is a relevant consideration in the therapeutic management of these patients.

These studies highlight the need for and importance of well-designed clinical trials that have sufficient power to detect differences in therapeutic efficacy that may be present between males and females, as distinct hormonal, genetic and immunological differences are documented between the sexes. It is critical that clinical trials are designed with careful consideration of these underlying sex-specific biological traits, as these likely have important relevance for personalized treatments.

## 8. Conclusion

In summary, a myriad of inter-related factors drive the female sex-bias that is evident in many autoimmune diseases, including pSD. Studies directed at understanding the underlying pathways that contribute to the profound female sex-bias observed in pSD will allow for the design of targeted therapies that may be tailored in accordance with the patient's biological sex for maximal efficacy.
